# “From misdiagnosis to precision medicine: strengthening pediatric neuro-oncology care in LMICs toward equitable pLGG outcomes”

**DOI:** 10.3389/fonc.2026.1895650

**Published:** 2026-08-26

**Authors:** Lorena V. Baroni, Nichoans B. George, Erin E. Crotty, Eric Bouffet, Mohamed S. Abdelbaki

**Affiliations:** 1 Division of Oncology, Hospital J. P. Garrahan, Buenos Aires, Argentina; 2 Division of Hematology, Oncology, Bone Marrow Transplant & Cellular Therapy, Department of Pediatrics, Seattle Children’s Hospital, University of Washington, Seattle, WA, United States; 3 Division of Hematology/Oncology, Hospital for Sick Children, Toronto, ON, Canada; 4 Division of Pediatric Hematology, Oncology and Bone Marrow Transplant, St. Louis Children’s Hospital, Washington University School of Medicine, St. Louis, MO, United States

**Keywords:** diagnostic discrepancy, global health equity, low- and middle-income countries (LMICs), MAPK-pathway inhibitors, pediatric low-grade glioma

## Abstract

Pediatric brain tumors—particularly pediatric low-grade gliomas (pLGG)—represent a major survivorship inequity between high-income countries (HICs) and low- and middle-income countries (LMICs). While pLGG is highly curable in optimal-resource settings, outcomes in LMICs remain suboptimal due to delays in diagnosis, limited neurosurgical capacity, restricted access to molecular testing, and significant variability in pathology accuracy and treatment accessibility. Data from international pathology review programs highlight a major diagnostic challenge: misclassification rates of 18–32% for pediatric CNS tumors, with discrepancies often driven by limited neuropathology expertise and lack of essential tools such as immunohistochemistry and molecular profiling. Studies from both global outreach networks and single-institution reviews consistently demonstrate that brain tumors account for the majority of major disagreements, and that molecular markers such as BRAF alterations and H3 mutations are critical for accurate diagnosis and risk stratification. These gaps directly affect treatment decisions and outcomes. At the same time, pLGG treatment paradigms are shifting with the development of oral targeted MAPK-pathway inhibitors, which deliver high response rates with favorable toxicity profiles. These agents hold unique promise for LMIC settings because they avoid the need for prolonged intravenous chemotherapy and reduce dependence on central lines and high-intensity supportive care. However, widespread adoption is limited by cost, regulatory barriers, absence from essential drug lists, and lack of molecular diagnostics required to select patients who would benefit most. This review synthesizes evidence from the evolving pLGG diagnostic and therapeutic landscape, and applies the Global Initiative for Childhood Cancer (GICC) CureAll framework to pediatric neuro-oncology care in LMICs. Key strategies include strengthening pathology accuracy through neuropathology partnerships, telepathology, and essential immunohistochemical/molecular panels; as well as integrating pLGG into national cancer control plans, enabling access to targeted therapies through differential pricing and pooled procurement, and building regional molecular diagnostic hubs. Together, these initiatives can reduce diagnostic errors, expand access to precision medicine, and move LMICs closer to equitable outcomes for children with pLGG.

## Background

Within the contemporary global oncology paradigm, an individual’s geographic location—frequently characterized as their “postal code”—exerts a more deterministic influence on patient outcomes than their inherent “genetic code” (1, 2). This survival disparity is particularly pronounced in the context of pediatric low-grade gliomas (pLGG). In high-income countries (HICs), pLGG is largely classified as an indolent, chronically manageable brain tumor with a 10-year OS rate in HICs consistently exceeding 90% (3, 4). Modern therapeutic advancements, such as the use of MEK inhibitors, have further solidified this status; for instance, recent long-term clinical outcomes from selumetinib trials demonstrate 5-year overall survival (OS) rates of 100% for patients with *KIAA1549-BRAF* fusions or *BRAF V600E* mutations (5). Conversely, in low- and middle-income countries (LMICs), these neoplasms remain a substantial contributor to pediatric morbidity and mortality (6, 7).

This profound inequity is not fundamentally attributable to innate biological heterogeneity; rather, it is precipitated by several factors, including systemic structural impediments to timely diagnostic identification and therapeutic access (8, 9). Global data indicate a direct relationship between annual governmental health-care expenditure and childhood cancer survival, with over 90% of childhood cancer deaths occurring in resource-limited settings where health-care systems are often underfunded (10). Diagnostic bottlenecks are further exacerbated by critical limitations in staffing and specialized resources. Comparative needs assessments have highlighted significant disparities in histopathology, where major referral centers in LMICs may operate with fewer specialized neuropathologists and lack the essential immunohistochemistry and molecular diagnostic markers (e.g., FISH probes) standard in HICs (11).

Furthermore, even when a diagnosis is established, treatment is frequently hindered by significant financial limitations. One-third (32%) of conventional chemotherapeutic drugs deemed essential by the WHO are only available in many LMICs through full out-of-pocket expenditure, placing families at high risk for catastrophic health spending (12). Consequently, as the World Health Organization (WHO) Global Initiative for Childhood Cancer (GICC) targets a 60% global survival rate by 2030, the mitigation of these unique diagnostic and therapeutic challenges associated with pLGG is a critical imperative for the advancement of global health equity (13, 14). The GICC CureAll framework serves as a tool to operationalize strategies to achieve global equity and work toward the overarching 2030 goal (14). Analyzing innovations in pLGG care within this framework can help to prioritize next steps for implementation.

## Review methodology

To synthesize the evolving evidence surrounding the diagnostic and therapeutic landscape for pLGG, we performed a targeted literature review. This methodology was structured to identify peer-reviewed publications investigating the nexus of precision medicine, resource-constrained environments, and pLGG clinical management.

### Search strategy

A rigorous search of major electronic databases, specifically PubMed/MEDLINE and Scopus, was executed to collate relevant literature. The retrieval strategy employed boolean combinations of primary concepts and keywords, including:

- Pediatric low-grade glioma
- Low- and middle-income countries
- Diagnostic discrepancy
- MAPK-pathway inhibitors
- Global health equity

### Inclusion and selection criteria

Selection prioritized data addressing the structural and clinical hurdles unique to pediatric neuro-oncology in LMICs. Inclusion criteria specifically targeted:

- Epidemiological evidence documenting significant baseline rates of diagnostic misclassification and discordance for pediatric CNS neoplasms in limited-resource settings.
- Landmark clinical trials and real-world implementation cohorts evaluating the safety, feasibility, and efficacy of targeted MAPK inhibitors.
- Infrastructural assessments for establishing telepathology partnerships and regionalized molecular diagnostic hubs.
- Policy research examining pooled procurement, differential drug pricing, and the integration of pLGG protocols into national cancer plans.

Studies not explicitly involving pediatric populations, CNS malignancies, or the socioeconomic and logistical realities of LMIC oncology were excluded.

### Data synthesis and framework application

Extracted findings were thematically synthesized to investigate the convergence of precision medicine and diagnostic stabilization. To operationalize these insights, the synthesis applies the WHO Global Initiative for Childhood Cancer (GICC) CureAll framework to the landscape of pediatric neuro-oncology care in LMICs.

### The diagnostic divide

The evolution of pediatric neuro-oncology has transitioned from purely histological and immunohistochemical classifications to a sophisticated molecular taxonomy (15, 16). The 2021 WHO Classification of CNS Tumors solidified the necessity of molecular profiling—such as identifying *BRAF* fusions or *H3* mutations—to provide accurate risk stratification (17, 18). However, for most LMICs, the diagnostic “gold standard” remains restricted to basic Hematoxylin and Eosin (H&E) staining and limited immunohistochemistry (19, 20). This infrastructure gap is particularly pronounced in Francophone Africa; a recent survey of 22 centers in the region revealed that nearly half (45%) lack access to immunohistochemistry, and an 86% have no access to molecular diagnostic tools, leaving patients in 19 of the 22 surveyed centers without the markers now considered essential for modern care (21).

This substantial degree of diagnostic uncertainty severely compromises clinical management, frequently resulting in the misidentification of tumor grade or type that drives inappropriate therapeutic decisions (22). Institutional experiences from regional referral centers in the Middle East further illuminate this diagnostic fragility, demonstrating that central nervous system neoplasms represent 71% of all significant diagnostic disagreements when compared to other pediatric solid tumors (23). These errors often involve the misidentification of tumor grade or type, directly leading to inappropriate treatment escalation or suboptimal management (24). In settings where neurosurgical capacity is already stretched thin, an incorrect diagnosis can lead to inappropriate surgical interventions or the administration of toxic, ineffective chemotherapy regimens, further straining the already limited healthcare resources (9, 20).

### The therapeutic paradox

pLGG are fundamentally characterized as a “single-pathway disease”, with constitutive activation of the mitogen-activated protein kinase (MAPK) signaling cascade occurring in 90% to 100% of all cases (4). This molecular landscape is predominantly driven by targetable *BRAF* alterations—specifically the *KIAA1549-BRAF* fusion (60–70%) and the *BRAF* V600E mutation (15–20%) (25–27), alongside *NF1* inactivation, *FGFR1* variants, and infrequent, yet highly actionable, fusions involving *NTRK*, *ALK*, or *ROS1* (<5%) (28, 29). Given this near-universal pathway reliance, the emergence of MAPK inhibitors, specifically MEK and BRAF inhibitors, represents a transformative inflection point for pediatric neuro-oncology (30). The clinical efficacy of these agents has been robustly validated in landmark trials, including CDRB436G2201, PBTC-029B and FIREFLY-1, which demonstrated sustained response rates in relapsed or refractory pLGG (5, 31, 32).

Historically, novel oncological therapies have been characterized as prohibitively complex for resource-constrained environments; however, targeted agents such as selumetinib and tovorafenib are well-suited for these settings due to their “infrastructure-light” delivery profile (33, 34). Unlike traditional cytotoxic chemotherapy, which mandates rigorous intravenous access, specialized infusion monitoring, and intensive supportive care for neutropenic complications, oral targeted therapies are typically managed in an outpatient setting (35). This decentralized model potentially reduces the necessity for central venous access, thereby minimizing the risk of line-associated complications such as bacteremia or febrile neutropenia, that might otherwise require inpatient management. Furthermore, the relatively manageable toxicity profile of MAPK inhibitors may support toxicity mitigation, potentially reducing hospitalizations in regions where tertiary care centers are remote or overextended (36). Real-world implementation data from LMICs substantiate these decentralized benefits; institutional cohorts from India and Jordan demonstrate that transitioning to oral MEK and BRAF inhibitors significantly reduces the frequency of hospital visits, increases school attendance, and yields high treatment satisfaction due to the avoidance of alopecia and severe myelosuppression, despite manageable, expected dermatological toxicities such as acneiform rash and paronychia (37, 38).

Nevertheless, a substantial implementation gap persists: despite their low-intensity administration requirements, these life-saving agents remain largely inaccessible due to prohibitive costs, a lack of inclusion on the World Health Organization (WHO) Model List of Essential Medicines, and a deficit in the molecular diagnostic infrastructure necessary to identify eligible patients (39–41). Research suggests a significant “diagnosis bias” exists in low-resource settings, where CNS tumors are likely underrepresented in official data due to limited access to advanced imaging, such as MRI, which is essential for identifying eligible patients. This phenomenon is robustly mirrored on a continental scale in Africa, where underdeveloped tracking frameworks and severe diagnostic infrastructure deficits drop the recorded incidence of pLGG far below global baselines, illustrating that patients are frequently missing from registries entirely, and therefore a larger number of patients are expected to be benefit from these life-altering agents compared to what is currently reported (42, 43).

## The precision diagnosis paradox

The clinical promise of MAPK-pathway inhibitors in resource-constrained environments introduces a profound operational contradiction, hereafter termed the “precision diagnosis paradox”. On one hand, oral targeted agents such as selumetinib or tovorafenib present a highly feasible, “infrastructure-light” therapeutic option for LMICs (33, 34). By eliminating the intensive clinical dependencies of conventional cytotoxic chemotherapy—such as permanent central venous access, strict inpatient supportive care, and high-frequency toxicity monitoring—these oral targeted therapies offer a highly decentralized, outpatient-manageable treatment model (35, 37). On the other hand, the clinical safety, regulatory approval, and therapeutic efficacy of these agents are strictly contingent upon precise molecular stratification (4, 17). Clinicians cannot safely or ethically prescribe allele-specific BRAF inhibitors (e.g., dabrafenib) or MEK inhibitors without first confirming the presence of specific driving alterations, such as the *KIAA1549-BRAF* fusion or the *BRAF* V600E mutation (17, 25, 31). This requirement creates a systemic bottleneck: a low-intensity therapeutic model cannot be deployed because its initiation is gated by highly complex, centralized, and costly diagnostic technologies that remain inaccessible to 80% of children living in resource-limited countries (11, 21).

To resolve this paradox and prevent the wholesale exclusion of LMIC patients from precision oncology, the global neuro-oncology community must reject binary diagnostic paradigms—which demand either comprehensive next-generation sequencing (NGS) or nothing—and instead champion pragmatic, tiered diagnostic pathways. Rather than waiting for the universal implementation of genomic sequencing or DNA methylation profiling, LMICs can utilize highly accessible, localized “bridging technologies” to guide targeted therapy safely (30, 33). For instance, immunohistochemistry (IHC) using the monoclonal antibody clone VE1 offers a highly sensitive, rapid, and cost-effective surrogate for detecting the *BRAF* V600E mutation (17, 33). Because IHC relies on basic pathology infrastructure that is already operational or easily scalable in regional referral centers, VE1 staining can immediately identify candidates for BRAF-targeted therapies without requiring molecular extraction (19, 22).

For the identification of the more prevalent *KIAA1549-BRAF* fusion, clinical networks can implement low-cost, targeted molecular platforms such as reverse transcription-polymerase chain reaction (RT-PCR) or single-plex fluorescence *in situ* hybridization (FISH) (30, 33). These targeted assays bypass the immense bioinformatics and financial burdens of NGS while maintaining high diagnostic accuracy. While regional molecular hubs and telepathology programs are progressively scaled under the WHO’s CureAll framework, these intermediate, assay-specific bridging strategies represent the only realistic pathway to align clinical capability with therapeutic opportunity, ensuring that patient selection remains both safe and equitable (14, 21, 44).

### Bridging the equity gap

**Figure 1:**
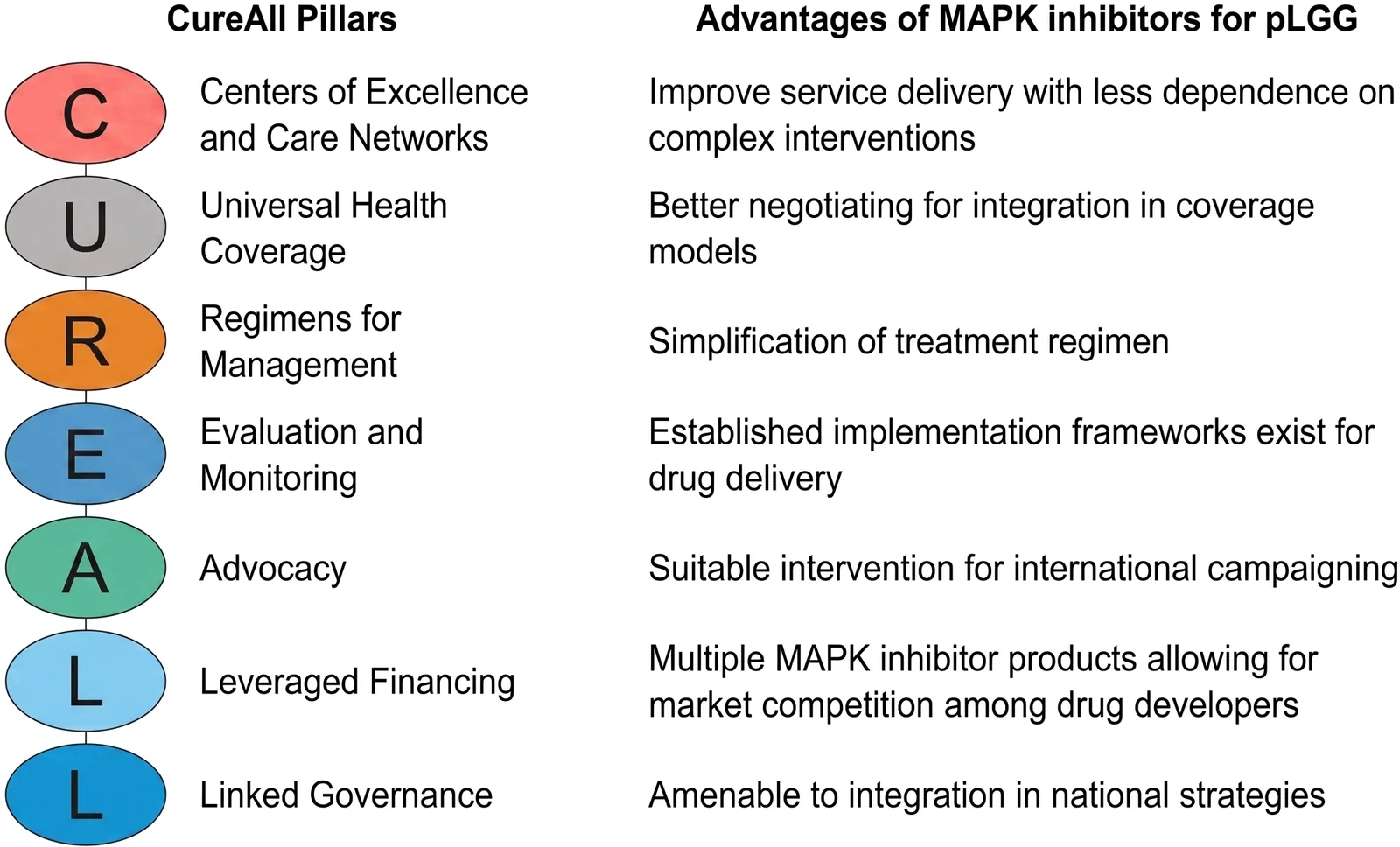
GICC CureAll framework mapping the systemic integration and infrastructure considerations for implementing MAPK inhibitors in pLGG across LMICs.

To translate current scientific advancements into a tangible clinical reality within LMICs, a comprehensive, multi-tiered strategic framework is required. The GICC utilizes the CureAll framework as its primary operational strategy to address global inequities in pediatric oncology with pLGG being one of six priority cancers. The CureAll approach is structured around four strategic pillars: centers of excellence and care networks, universal health coverage, regimens for management (standardized treatment protocols), and evaluation and monitoring. These pillars are supported by three core enablers—advocacy, leveraged financing, and linked governance—which ensure that childhood cancer is integrated into national health agendas and remains sustainable (14). The application of this framework to examine the role of MAPK inhibitors in LMIC settings is particularly useful (**Figure 1**).

This strategic alignment is increasingly reflected in the grassroots efforts of several LMICs to design and implement standardized, resource-stratified clinical guidelines specifically tailored to their healthcare ecosystems. For instance, collaborative capacity-building initiatives in Pakistan have demonstrated how establishing dedicated subspecialty pediatric neuro-oncology services and unified regional management protocols can drastically optimize clinical pathways, even when operating under severe radiotherapy or neurosurgical limitations (44, 45). Similarly, cooperative regional networks across Africa have initiated baseline mapping studies to systematically document pLGG outcomes and infrastructure gaps, establishing a data-driven foundation for the rollout of GICC-aligned national protocols (42). These national and regional efforts—further supported by real-world clinical evidence from pioneering institutional protocols in India and Jordan (37, 38)—prove that the systematic adaptation of standardized regimens is not only feasible but represents an indispensable prerequisite for improving survival rates and achieving diagnostic and therapeutic equity (8, 14). LMIC collaborative efforts for pLGG are summarized in **Table 1**.

**Table 1:** Summary of collaborative initiatives and clinical implementations for pLGG management in LMICs.

Region/country	Collaborative effort & clinical focus	Key implementation outcomes and impacts	Reference(s)
Pakistan	* Establishment of Dedicated Pediatric Neuro-Oncology Services (DPNOS). * Implementation of unified regional management protocols and cross-institutional memorandums of understanding to bypass local resource deficits.	* Expanded patient referral volumes. * Elevated the proportion of pediatric patients treated with curative intent. * Significantly improved patient overall survival rates.	(46–48)
Africa	* Multicenter baseline mapping studies across regional networks to document pLGG outcomes. * Diagnostic resource surveys across 22 centers in Francophone Africa.	* Documented critical baseline disparities, showing 45% of surveyed centers lacked IHC and 86% lacked molecular tools. * Established a data-driven baseline to guide the rollout of GICC-aligned national protocols.	(21, 42)
India and Jordan	* Real-world, institutional cohort applications of oral targeted MAPK pathway inhibitors (MEK and BRAF inhibitors)	* Significantly reduced hospital visit frequencies and minimized inpatient stays. * Elevated school attendance and treatment satisfaction. * Avoided toxicities like alopecia and severe myelosuppression, maintaining manageable dermatological profiles.	(37, 38)
Lebanon	* Retrospective single-institution reviews of pediatric oncology pathology.	* Demonstrated that central nervous system tumors carry the highest rate of major diagnostic discrepancies.	(23)
Global / Regional Networks	*Multi-center international pediatric neuro-oncology teleconferences and global tumor boards.	*Optimally altered or refined clinical management decisions in approximately 32% of complex cases.	(22, 45, 49, 50)

Beyond MAPK inhibitors as a specific innovation in pLGG care, the persistent survival disparity in pediatric neuro-oncology is a failure of clinical implementation, resource distribution and severe diagnostic latency (49). The mitigation of diagnostic volatility through formalized neuropathology twinning and telepathology programs is a primary requirement (22, 50). By establishing collaborative partnerships between LMIC centers and HIC departments, real-time expert consultation and secondary reviews can be institutionalized. Crucially, the expansion of bilateral twinning initiatives into multi-center regional teleconferences has demonstrated that expert second opinions can significantly optimize clinical management, altering or refining treatment modalities in 32% of complex cases—an intervention of paramount importance given that the majority of LMIC oncologists manage these cases without formal subspecialty training in neuro-oncology. This virtual collaborative paradigm is further enhanced by large-scale, international tumor boards that successfully bridge resource gaps, enhance diagnostic precision, and guide therapeutic decision-making; notably, a substantial proportion of consultations in these global forums are directed toward pLGG (23.4%) and neurofibromatosis-associated tumors (32.7%) (46, 51). Furthermore, diagnostic standardization must be prioritized through the procurement of “minimum essential” immunohistochemical panels—incorporating markers such as GFAP, Ki-67, *BRAF* V600E, and *H3K27M*—to serve as the baseline diagnostic threshold (17). To explicitly address early diagnostic delays before patients reach these centers, incorporating frontline diagnostic heuristics like the “LOW-OR-PAY” clinical mnemonic or public-professional awareness frameworks—modeled after the UK’s “HeadSmart” campaign, which successfully reduced the median total diagnostic interval from 14 to 6.7 weeks—is vital to accelerating the path to neuroimaging (47).

To overcome resource fragmentation, the adoption of a “Hub-and-Spoke” model is proposed, wherein centralized regional molecular diagnostic hubs concentrate technical expertise and utilize economy of scale to reduce the per-test costs of high-complexity assays, including Polymerase Chain Reaction (PCR) and Next-Generation Sequencing (NGS) (30, 45). Such structured networks and specialized care delivery models mirror the documented success of dedicated pediatric neuro-oncology services (DPNOS) in LMIC settings. This institutional paradigm shift is compellingly demonstrated by a landmark single-institution experience in Pakistan, where the formal establishment of a DPNOS dramatically optimized clinical metrics (45). Empirical data indicate that transitioning from generalized oncology care to dedicated subspecialty teams—integrated with formalized cross-institutional networks and memorandums of understanding to circumvent local infrastructure deficits such as radiotherapy or neurosurgical limitations—can dramatically expand referral volumes, elevate the proportion of patients treated with curative intent, and significantly improve overall survival rates (44, 48).

To operationalize these specialized services, we propose a tri-phased, resource-stratified framework for progressive physical and clinical infrastructure development aligned with GICC principles:

- Phase 1 (Diagnostic Stabilization): Centers prioritize neutralizing diagnostic latency by standardizing pediatric neuroimaging (42), instituting routine tumor biopsies to eliminate presumptive diagnoses (21, 42), and validating low-cost IHC surrogates—such as the BRAF V600E (VE1) antibody—using existing, scalable pathology laboratories (17, 33).
- Phase 2 (Multidisciplinary Consolidation): Institutions upgrade neurosurgical operating suites, establish standardized pediatric neuro-intensive care protocols to mitigate perioperative risk (9, 43), and scale international twinning and telepathology programs to secure continuous expert second opinions (22, 46, 50).
- Phase 3 (Precision Integration): Teams integrate low-cost, targeted molecular assays locally (such as RT-PCR or single-plex FISH for *KIAA1549-BRAF* fusions) (30, 33), while utilizing centralized regional hubs for high-complexity genomic sequencing and DNA methylation profiling (30, 45).

**Figure 2:**
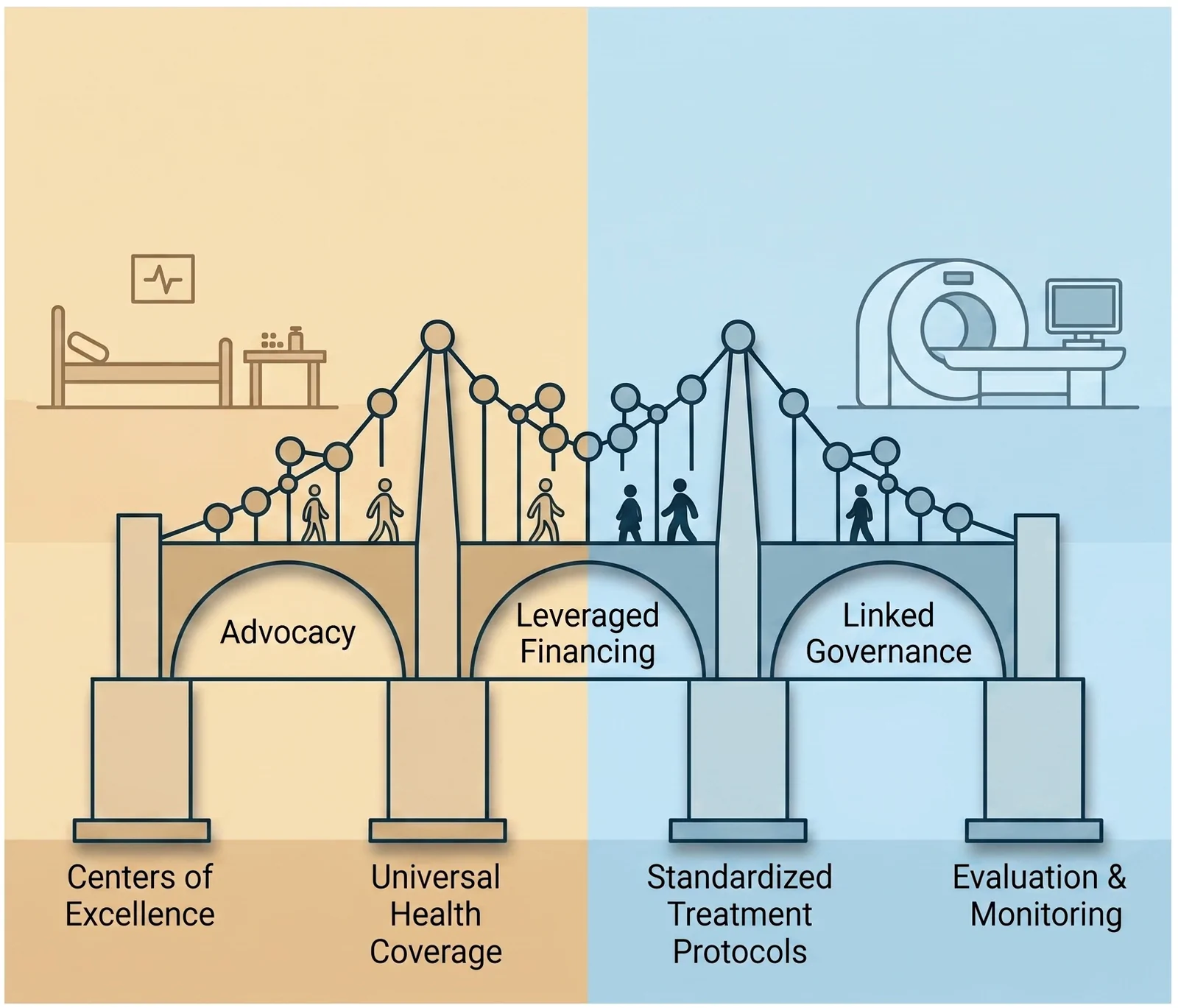
Conceptual adaptation of the CureAll framework for pLGG in LMICs.

The transition toward precision medicine requires a robust strategy for sustainable pharmacotherapy access. We advocate for the utilization of pooled procurement mechanisms, leveraging regional economic blocs to aggregate demand and negotiate equitable pricing for targeted inhibitors (30, 47). This economic strategy must be complemented by rigorous policy integration, specifically the inclusion of pediatric-specific targeted therapies within National Cancer Control Plans (NCCPs) and National Essential Medicines Lists to facilitate government-subsidized access and long-term sustainability (47) (**Figure 2**).

## Structural impediments and implementation hurdles for proposed strategic interventions

Although the systemic integration of specialized neuropathology boards and molecular technologies presents a transformative opportunity to enhance clinical outcomes, these initiatives are precipitated by significant structural impediments and longitudinal implementation challenges. Specifically, the scalability of complex molecular infrastructure mandates consistent financial prioritization, robust cold-chain logistics, and perpetual technical capacity building, which may saturate the operational capacity of resource-limited healthcare environments. Moreover, a disproportionate emphasis on centralized, high-technology paradigms may inadvertently intensify regional inequities if foundational primary and secondary care tiers remain under-resourced. Calibrating the adoption of precision modalities with the stabilization of essential histopathology services—including standardized H&E protocols and institutionalized referral frameworks—is a critical imperative to mitigate unsustainable fiscal and operational burdens.

### Final considerations and future directions

The survival disparity for children diagnosed with pLGG represents a profound and remediable global health injustice. Addressing this inequity requires a shift from conventional treatment paradigms toward a specialized, multi-sectoral strategy tailored to the infrastructure of LMICs. Crucially, contemporary diagnostic frameworks—such as the 2021 WHO Classification of Tumors of the Central Nervous System—increasingly predicate definitive disease categorization on sophisticated molecular and genomic alterations (17). By defaulting to a generic “Not Otherwise Specified” (NOS) designation when such advanced profiling is unavailable, these international guidelines inadvertently overlook the stark material and financial realities of resource-limited settings, effectively codifying a diagnostic disconnect between high- and low-resource environments. To counteract this institutional marginalization, the global oncological community must prioritize diagnostic precision through the optimization of accessible, baseline immunohistochemical panels alongside international neuropathology consortia, while simultaneously leveraging the logistical advantages of targeted oral therapeutics to significantly attenuate the survival gap (13, 15).

Future efforts must focus on the formal integration of these molecularly-driven frameworks into national health policies and the stabilization of pharmaceutical supply chains through innovative procurement models. Such initiatives transcend the improvement of individual clinical outcomes; they constitute a fundamental advancement toward the democratization of precision oncology, ensuring that the benefits of genomic medicine are accessible to all pediatric patients, irrespective of geographic or socioeconomic constraints (2, 41).
